# The N‐Bromo‐Hammick Intermediate

**DOI:** 10.1002/chem.202502434

**Published:** 2025-09-26

**Authors:** Virinder Bhagat, J. Philipp Wagner

**Affiliations:** ^1^ Institut für Organische Chemie Eberhard Karls Universität Tübingen Auf der Morgenstelle 18 Tübingen 72076 Germany; ^2^ Institut für Organische und Analytische Chemie Universität Bremen Leobener Straße 7 28359 Bremen Germany

**Keywords:** computational chemistry, hydrogen activation, matrix isolation, N‐heterocyclic carbenes

## Abstract

We report the characterization of bromopyridin‐2‐ylidene, the N‐bromo‐Hammick intermediate, isolated in solid neon at 4.4 K. Computations (B2PLYP, NEVPT2), IR, and UV/vis spectroscopy characterize this intermediate as a singlet carbene with a vacant σ*‐type frontier orbital. This unusual electronic structure (σ^2^σ*^0^) allows the carbene to isomerize to its more stable pyridine tautomer *via* a planar transition state, avoiding the deviation from planarity seen in other singlet carbenes. The compound's reaction with molecular hydrogen faces a minor but prohibitive barrier (8.4 kcal mol^−1^) at the low temperatures of the experiment.

## Graphical abstract

Spectroscopic and computational analysis uncovers the σ^2^σ*^0^ electronic structure of the N‐bromo‐Hammick intermediate, a singlet N‐heterocyclic carbene isolated in solid neon, whose back‐isomerization to bromopyridine proceeds *via* a planar transition state.

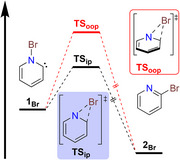

## 1 Introduction

Pyridinylidenes constitute an intriguing class of N‐heterocyclic carbenes that feature only a single nitrogen atom, endowing them with superior σ‐donor and π‐acceptor properties.^[^ ^1^ ^]^ Hammick first postulated that these carbenes form during the decarboxylation of α‐picolinic acid, where they can subsequently react with carbonyl compounds.^[^ ^2^ ^]^ Consequently, these species are commonly referred to as Hammick intermediates. Various metal complexes of Hammick intermediates have been synthesized through methods such as thermolysis of pyridinium carboxylates,^[^ ^3^ ^]^ oxidative addition,^[^ ^4^ ^]^ and C − H activation reactions,^[^ ^5^ ^]^ and their application in catalysis has been studied.^[^ ^6^ ^]^ Due to its highly transient nature, the free parent Hammick intermediate has only been observed using mass spectrometry.^[^ ^7^ ^]^

**Scheme 1:**
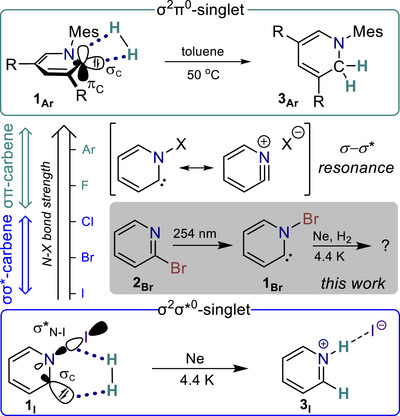
Electronic structure dependence of pyridinylidenes on the bond strength between the nitrogen atom and its substituent [N − X, (X = I, Br, Cl, F, Ar)], resulting in two groups of pyridinylidenes: σσ*‐carbenes and σπ‐carbenes. R = 2,6‐Me_2_C_6_H_3_.

Strategies to stabilize these elusive carbenes have been devised by introducing amino groups at the ring and bulky substituents at the nitrogen atom.^[^ ^8^ ^]^ However, even the tri‐aryl substituted free pyridinylidene **1_Ar_** could not be isolated in pure form (Scheme 1).^[^ ^9^ ^]^ A breakthrough occurred only recently with the crystallization of the sterically crowded benzo[h]isoquinolin‐1‐ylidene.^[^ ^10^ ^]^ Given their fleeting nature, it is even more surprising that our lab has recently discovered a facile preparation of an N‐iodinated version of the Hammick intermediate, **1_I_**, by simple UV photolysis of 2‐iodopyridine in solid neon.^[^ ^11^ ^]^ The resulting carbene is stabilized by an in‐plane resonance interaction of the carbene's σ_C_ orbital and the antibonding orbital of the N − I bond, σ*_N−I_ (Scheme 1). Hence, both orbitals become frontier orbitals, rendering this pyridinylidene a σ^2^σ*^0^ singlet ground state carbene.

The unusual electronic structure of the halogenated Hammick intermediate **1_I_** introduces an unprecedented reactivity, particularly in its interaction with molecular hydrogen. While conventional σ^2^π^0^ singlet carbenes, such as the aryl‐substituted pyridinylidene **1_Ar_**, typically activate H_2_ *via* a non‐least‐motion π‐approach,^[^ ^12^ ^]^ carbene **1_I_** follows an in‐plane addition mode, predominantly yielding pyridinium iodide in a neon matrix (Scheme 1).^[^ ^11^ ^]^ This highlights the broad reactivity range of pyridinylidenes, which is dictated by their electronic structure. Our computational studies reveal that this electronic structure landscape can be tuned by varying the nitrogen substituent: weak N − X bonds (e.g., X = I) lead to the σ^2^σ*^0^ configuration, while strongly bound substituents (e.g., X = Ar) favour a σ^2^π^0^ ground state (Scheme 1).^[^ ^11^ ^]^ In this work, we set out to explore a more intermediate regime between these competing electronic structures by investigating the *N‐bromo Hammick intermediate*, **1_Br_** (Scheme 1). Our goal is to understand its reactivity, particularly in terms of isomerization and hydrogenation, in comparison to the two limiting cases.

## 2 Results and Discussion

**Figure 1:**
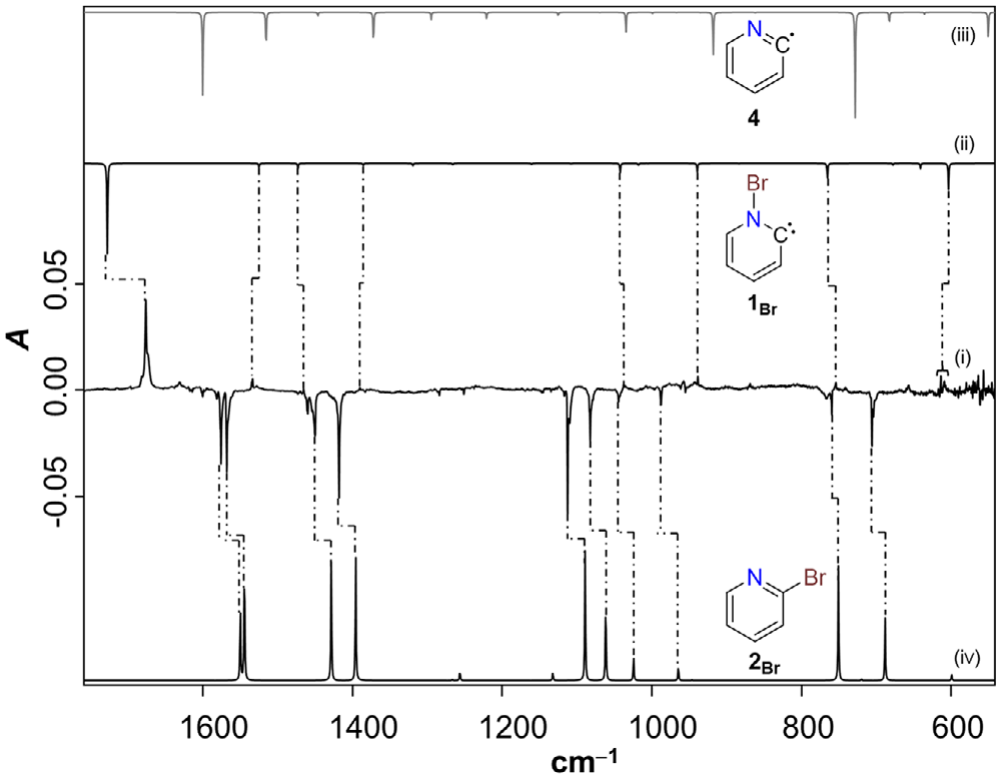
(i) Difference IR spectrum after irradiation of a neon matrix containing **2_Br_** for 5 min with λ = 254 nm. (ii) Anharmonic IR spectrum (fundamental bands only) of **1_Br_** computed at the B2PLYP‐D3/def2‐TZVPP level of theory; (iii) and (iv) are computed harmonic spectra (scaled, scaling factor: SF: 0.9605) of **4** and **2_Br_**, respectively (B2PLYP‐D3/def2‐TZVPP).

In order to prepare the N‐Bromo‐Hammick intermediate **1_Br_**, we co‐deposited its alleged precursor 2‐bromopyridine **2_Br_** with a large excess of neon gas onto a cold CsI window continuously maintained at 4.4 K. Following this step, we irradiated the deposited matrix with λ  =  254 nm for five minutes, which led to a bleaching of the IR bands associated with **2_Br_**, as shown in the difference spectrum in Figure 1 (i). The assignment of the decreasing bands to **2_Br_** was confirmed by comparison with the respective scaled harmonic IR spectrum computed at the B2PLYP‐D3/def2‐TZVPP level of theory [Figure 1 (iv)].

As for the products formed, the 2‐pyridyl radical **4**, which could result from a competing photochemical N − Br bond homolysis, does not account for the observed IR bands. This conclusion is supported by comparisons with both literature data and the computed IR spectrum of **4** [Figure 1 (iii)].^[^ ^13^ ^]^ In contrast, the increasing IR bands in Figure 1 (i), located at 1677, 1534, 1466, 1391, 1038, 939, 754, 614, and 609 cm^−1^, are consistent with those predicted for the expected photoproduct **1_Br_**. These bands exhibit excellent agreement with the computed anharmonic IR spectrum of the N‐bromo‐Hammick intermediate [Figure 1 (ii)], strongly supporting its formation. Accordingly, we assign carbene **1_Br_** as the major product of the photochemical isomerization of pyridine **2_Br_**. Pyridinylidene **1_Br_** was found to be highly photolabile, as its IR bands vanished and the precursor **2_Br_** reappeared upon ten minutes of sunlight exposure (Figure S1) − a behaviour previously observed for the N‐iodo‐Hammick intermediate **1_I_**.^[^ ^11^ ^]^

**Figure 2:**
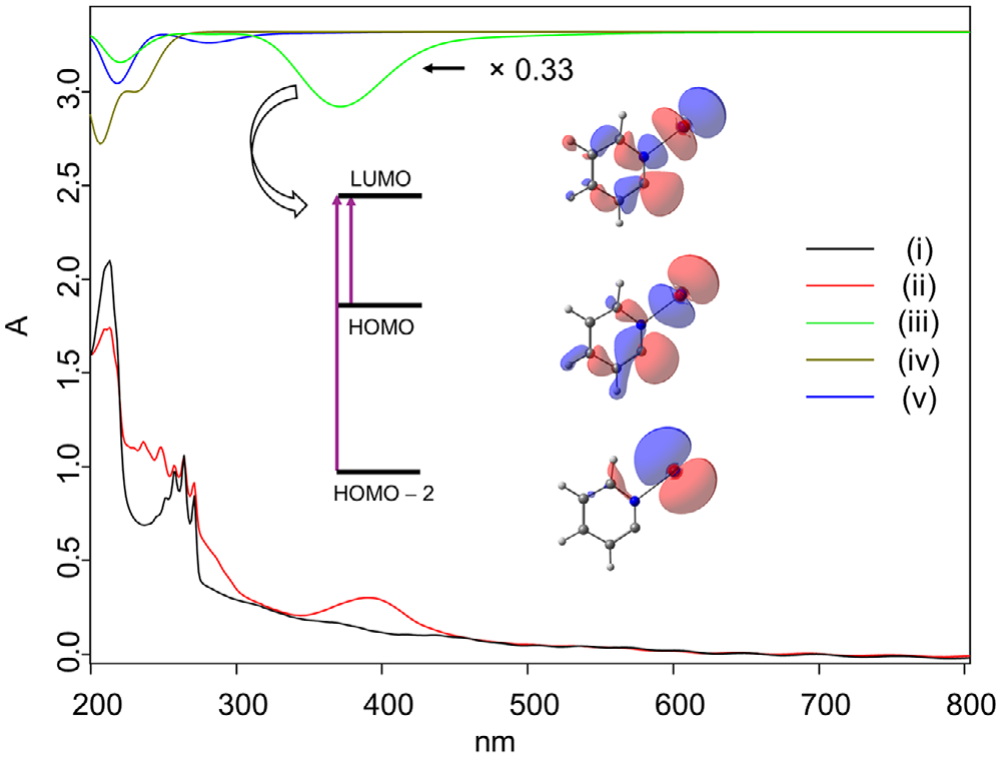
(i) UV/vis spectrum after co‐deposition of precursor **2_Br_** with a large excess of argon for 15 min. (ii) Irradiation of the matrix for 4 min with λ = 254 nm; (iii), (iv), and (v) show the computed UV/vis spectra of **1_Br_**, **2_Br_**, and **4**, respectively, at the CAM‐B3LYP/def2‐TZVPP//B2PLYP‐D3/def2‐TZVPP level of theory.

We further characterised carbene **1_Br_** using UV/vis spectroscopy under similar experimental conditions, apart from the utilization of argon as the matrix host. The resulting spectrum [Figure 2 (ii)] after irradiation with λ  =  254 nm shows a broad emerging band centred at 391 nm. This absorption feature in the visible region of the spectrum supports the formation of **1_Br_** with its close‐lying frontier orbitals. Moreover, the computed spectrum of **1_Br_** [CAM‐B3LYP/def2‐TZVPP, Figure 2 (ii)] displays a convincing agreement with the experimental spectrum. Thus, the broad band in the experiment is assigned to an electronic transition with major contributions from the σ_C_ HOMO to σ*_N−I_ LUMO electron transfer (*cf*. Figure 2). Akin to our infrared experiments, the band at 391 nm is photolabile and vanishes upon exposure of the matrix to sunlight for four minutes, resulting in the regeneration of precursor **2_Br_** (Figure S2). Therefore, we conclude that bromopyridin‐2‐ylidene **1_Br_** is the major product of the photochemical isomerization of 2‐bromopyridine, exhibiting a σ^2^σ*^0^ electron configuration comparable to that of the previously synthesized **1_I_**.

The pyridinylidenes **1_Br_** and **1_I_** display qualitatively similar IR and UV/vis spectra, that are accompanied by a blue‐shift of the respective strongest observed spectral transitions.^[^ ^11^ ^]^ In the IR spectrum of **1_Br_**, the most intense band at 1677 cm^−1^ primarily corresponds to a stretching vibration of the bond between the nitrogen atom to the carbenic center. This feature is absent in pyridinylidenes with a σ^2^π^0^ electron configuration (Figure S6) and can therefore be seen as a signature of the σ−σ* resonance interaction with its triply bound mesomeric structure (*cf*. Scheme 1). The band is blue‐shifted by 26 cm^−1^ in comparison to **1_I_**, indicating a stronger resonance interaction in the bromo case in accordance with our natural bond orbital analysis (Figure S8). We further note that the theoretical description of the C − N stretching vibration is particularly sensitive to multireference effects explaining the discrepancy of experiment and theory for this particular band (*cf*. Figure 1, Table S1). The stronger resonance interaction in **1_Br_** is accompanied by a larger energy gap between the σ_C_ and σ*_N−Br_ frontier orbitals causing the 0.2 eV blue‐shift of the strongest band in the UV/vis spectrum in comparison to **1_I_**. This observed trend in electronic and vibrational spectroscopy is continued by the N‐chloro Hammick intermediate **1_Cl_**, as indicated by our computational data in Figures S6 and S7.

**Figure 3:**
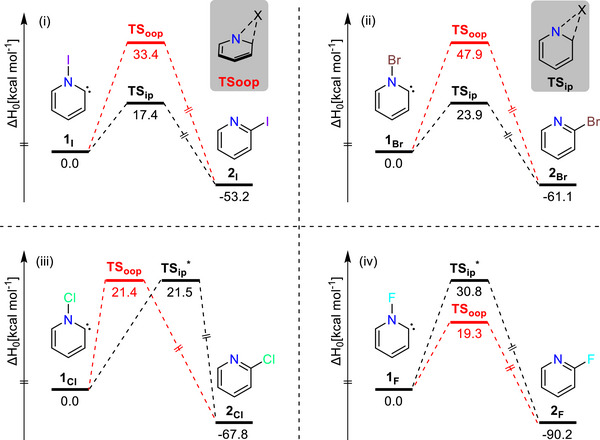
Potential energy surfaces corresponding to the [1,2]X [X = I (i), Br(ii), Cl(iii), and F(iv)] shifts in different Hammick intermediates (**1_I_**, **1_Br_**, **1_Cl_**, and **1_F_**) *via* in‐plane and out‐of‐plane reaction pathways calculated at the NEVPT2/def2‐TZVPP//B2PLYP‐D3/def2‐TZVPP level of theory.

In addition to its impact on spectroscopy, the carbene's σ^2^σ*^0^ electron configuration gives access to new reaction pathways, which becomes evident, for instance, in its tautomerization reaction to the more stable pyridine isomer. Usually, such a 1,2‐migration reaction is orbital symmetry forbidden,^[^ ^14^ ^]^ and, therefore, singlet carbenes deviate from planarity to exploit more favourable orbital interactions with the vacant π_C_ orbital.^[^ ^15^ ^]^ The pronounced σ_C_−σ*_N−Br_ resonance interaction leads to a weakening of the N − Br bond, so that an in‐plane isomerization becomes possible. To gauge the electronic structure dependence of this rearrangement, we computed the reaction barriers for the competing in‐plane (**TS_ip_**) and out of plane saddle points (**TS_oop_**) for the series of the halogens (I, Br, Cl, and F) with increasing N − X bond strengths (Figure 3).

**Figure 4:**
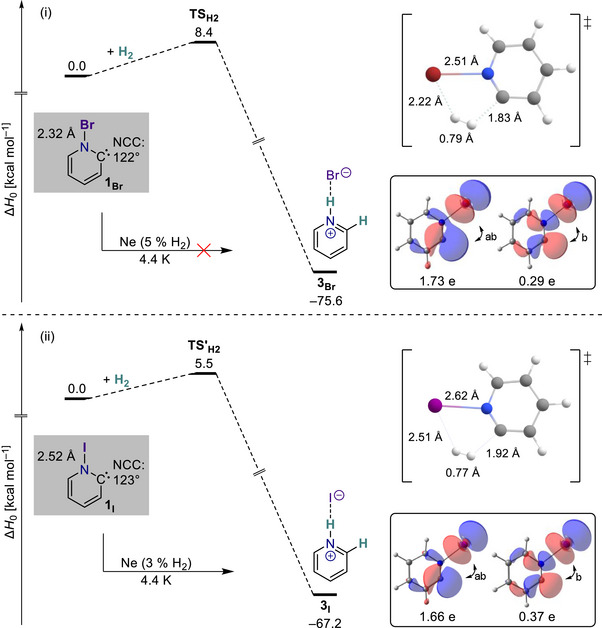
Comparison of the potential energy surfaces for the hydrogenation reaction of the N‐Bromo‐Hammick intermediate **1_Br_** (i) and the N‐Iodo‐Hammick intermediate **1_I_** (ii). The PESs were calculated at the NEVPT2/def2‐TZVPP//B2PLYP‐D3/def2‐TZVPP level of theory with an active space of 12 electrons and 11 orbitals (see Figure S5). Inset1 (top parts): Calculated geometries of the transition states for the hydrogenation of **1_Br_** and **1_I_** at B2PLYP‐D3/def2‐TZVPP level of theory. Inset2 (bottom parts): Selected natural orbitals and their electron occupation numbers of **1_Br_** and **1_I_** at their respective B2PLYP‐D3/def2‐TZVPP geometries.

We find that **TS_oop_** is structurally characterised by shorter N − X bonds in comparison to **TS_ip_**, which we associate with the bond weakening effect of the σ_C_−σ*_N−X_ resonance interaction (Figure S3). Furthermore, all saddle points were found to be of first order, except for the in‐plane isomerization pathways of carbenes **1_Cl_** and **1_F_**, which correspond to second‐order saddle points and are therefore avoided. Based on the data presented in Figure 4, carbenes **1_I_** and **1_Br_** preferentially undergo the [1,2]X‐shift *via* the in‐plane mode, whereas carbene **1_Cl_** is energetically almost indifferent. In contrast, the N‐fluoro substituted Hammick intermediate **1_F_** favours the out‐of‐plane mode for its [1,2]F‐shift reaction. This is a consequence of the altered electronic structure of carbene **1_F_** featuring the conventional σ^2^π^0^ configuration, which makes **TS_ip_** less favorable.^[^ ^11^ ^]^ We note that the simplest Hammick intermediate (X = H) also favours an isomerization pathway to pyridine that remains largely planar.^[^ ^7^ ^]^ However, this process may be better understood as an acid–base reaction, given the high basicity of pyridinylidene. In contrast, methyl‐ and phenyl‐substituted carbenes still proceed traversing **TS_oop_** (Figure S3).

The in‐plane σ* approach addition of molecular hydrogen is another hallmark reaction of the new σ^2^σ*^0^‐type carbenes (*cf*. Scheme 1).^[^ ^11^ ^]^ This compels us to investigate the reaction of **1_Br_** with dihydrogen in comparison to the previously experimentally realized reaction of **1_I_**. The electronic structure of **1_Br_** is similar to that of **1_I_** in a complete active space self‐consistent field (CASSCF) treatment, but the σ‐ and σ*‐type frontier orbitals feature electron occupation numbers closer to the expected integer values (2 and 0, respectively; Figure 4). In agreement with the stronger N − Br bond, our NEVPT2 computations suggest that the kinetic barrier for hydrogenation increases by approximately 3 kcal mol^−1^, relative to that of **1_I_**. Hence, the addition of dihydrogen to **1_Br_** is expected to be slower than in case of **1_I_** despite the higher driving force (Figure 4).

To investigate the reaction experimentally, precursor **2_Br_** was co‐deposited on a cold CsI window with an excess of neon gas doped with 5% H_2_. Following this, we irradiated the matrix, which resulted in the bleaching of IR bands associated with **2_Br_** and the observation of new IR bands, as shown in the difference spectrum in Figure S4. When comparing the experimental bands assigned to **1_Br_** with the new IR bands observed (Figure S4), it can be safely concluded that the irradiation only resulted in the formation of Hammick intermediate **1_Br_** and no reaction with H_2_ whatsoever had occurred**_._** This behavior is in stark contrast to the case of **1_I_**, which underwent a facile reaction with H_2_, forming pyridinium iodide **3_I_** (Scheme 1).^[^ ^11^ ^]^ The reduced diradical character of **1_Br_** might also preclude the quantum mechanical tunneling facilitated hydrogen atom abstraction pathway, which was previously predicted from instanton theory computations for the low temperature regime.^[^ ^11^, ^16^ ^]^

## 3 Conclusions

In this study, we have successfully isolated and characterized the N‐bromo‐Hammick intermediate **1_Br_** using IR and UV/vis spectroscopy. Electronically, **1_Br_** features σ/σ*‐type frontier orbitals, similar to those of the previously reported carbene **1_I_**, but with a stronger N–Br bond. Computational studies indicate that **1_Br_** favors in‐plane modes for its isomerization and H_2_ activation reactions, consistent with its σ^2^σ*^0^ electronic structure. However, **1_Br_** exhibits a higher kinetic barrier for the H_2_ addition reaction compared to **1_I_**, and thus shows no reactivity with the diatomic under cryogenic conditions at 4.4 K.

## Conflict of Interest

The authors declare no conflict of interest.

## Supporting information



Supporting Information

## Data Availability

The data supporting this article have been included as part of the Supporting Information.
