# Supplementary material for: The N‐Bromo‐Hammick Intermediate
Source: Chemistry. 2025 Sep 26;31(60):e02434. doi: 10.1002/chem.202502434 (PMC12559450; doi:10.1002/chem.202502434)
Supplement: Supplementary file 1 — Supporting Information [file CHEM-31-e02434-s001.pdf]

Supporting Information for

The N-Bromo-Hammick intermediate

Virinder Bhagat[a] and J. Philipp Wagner*[a,b]

Affiliations:

[a]Institut für Organische Chemie, Eberhard Karls Universität Tübingen, Auf der Morgenstelle 18, 72076 Tübingen, Germany
[b]Institut für Organische und Analytische Chemie, Universität Bremen, Leobener Straße 7, 28359 Bremen

*Corresponding author: jpw@uni-bremen.de

Table of Contents

1. Materials and Methods……2
2. Additional Spectroscopy and Computational Data……3
3. IR Band Data……12
4. Cartesian Coordinates and Energies……13
5. References……20

Materials and Methods

Matrix Isolation Experiments

Experiments were conducted under cryogenic conditions utilizing two matrix isolation systems, one for infrared (IR) spectroscopy and the other for ultraviolet and visible (UV/vis) studies. For IR studies, a SHI CKW-21A closed-cycle helium cryostat was employed, which reaches a minimum temperature of 4.4 K. An APD HC-2 closed-cycle helium cryostat was used for UV/vis experiments, with a minimum temperature of 6 K. In addition, pressures below 10^{-6} mbar were consistently maintained using an Edwards oil diffusion pump. The precursor 2-bromopyridine (BLD Pharm Ltd, 99.95%), along with a huge amount of matrix host, either neon or argon (Messer-Griesheim, 99.9999%), was co-deposited onto cold CsI or sapphire spectral windows. For the reaction with dihydrogen, the matrix host was doped with 3% H_2 (Westfalen, 99.999%). The sample holder containing 2-bromopyridine was externally immersed in a cold bath maintained at –40 °C during deposition. A consistent flow rate of the host matrix gases was maintained by an MKS mass flow controller. For the photochemical transformations described, a low-pressure mercury lamp (λ = 254 nm, PenRay) and a high-pressure mercury lamp (USHIO, USH-508S) were utilized. For analysis of the experimental results, a Bruker Vertex 70 spectrophotometer, with a resolution of 0.5 cm^{-1}, was used for IR measurements in the range of 4000–400 cm^{-1}, while UV/vis data were acquired with a PerkinElmer 1050 spectrophotometer.

Computational Details

Geometry optimizations of different species were performed employing the B2PLYP functional[1] in combination with Grimme's D3 correction[2] and the Karlsruhe def2-TZVPP basis set.[3] To characterize the optimized geometries as minima or first-order saddle points and to obtain zero-point corrections to electronic energies, vibrational frequency calculations using the harmonic approximation were carried out. Moreover, the vibrational frequencies were used to obtain the calculated IR spectrum of different chemical species, which were scaled with an optimized scaling factor.[4] The anharmonically corrected vibrational frequencies and IR spectra were computed using second-order vibrational perturbation theory (VPT2).[5] In addition, the refined energies of the stationary points were calculated with n-electron valence state perturbation (NEVPT2) theory without the frozencore approximation,[6] which was combined with the def2-TZVPP basis set. An active space of 10 electrons and 9 orbitals was used for the [1,2]X [X = I, Br, Cl, and F] shifts in different Hammick intermediates, while the active space was augmented by the additional σ and σ* orbitals of dihydrogen in case of the hydrogenation reactions (Fig. S5). DFT calculations were carried out using the Gaussian16 quantum chemistry package[7] and for the NEVPT2 calculations, ORCA 5.0.1[8] was employed. In addition, NBO 6.0 was used for the natural bond orbital analysis.[9]

Additional Spectroscopy and Computational Data

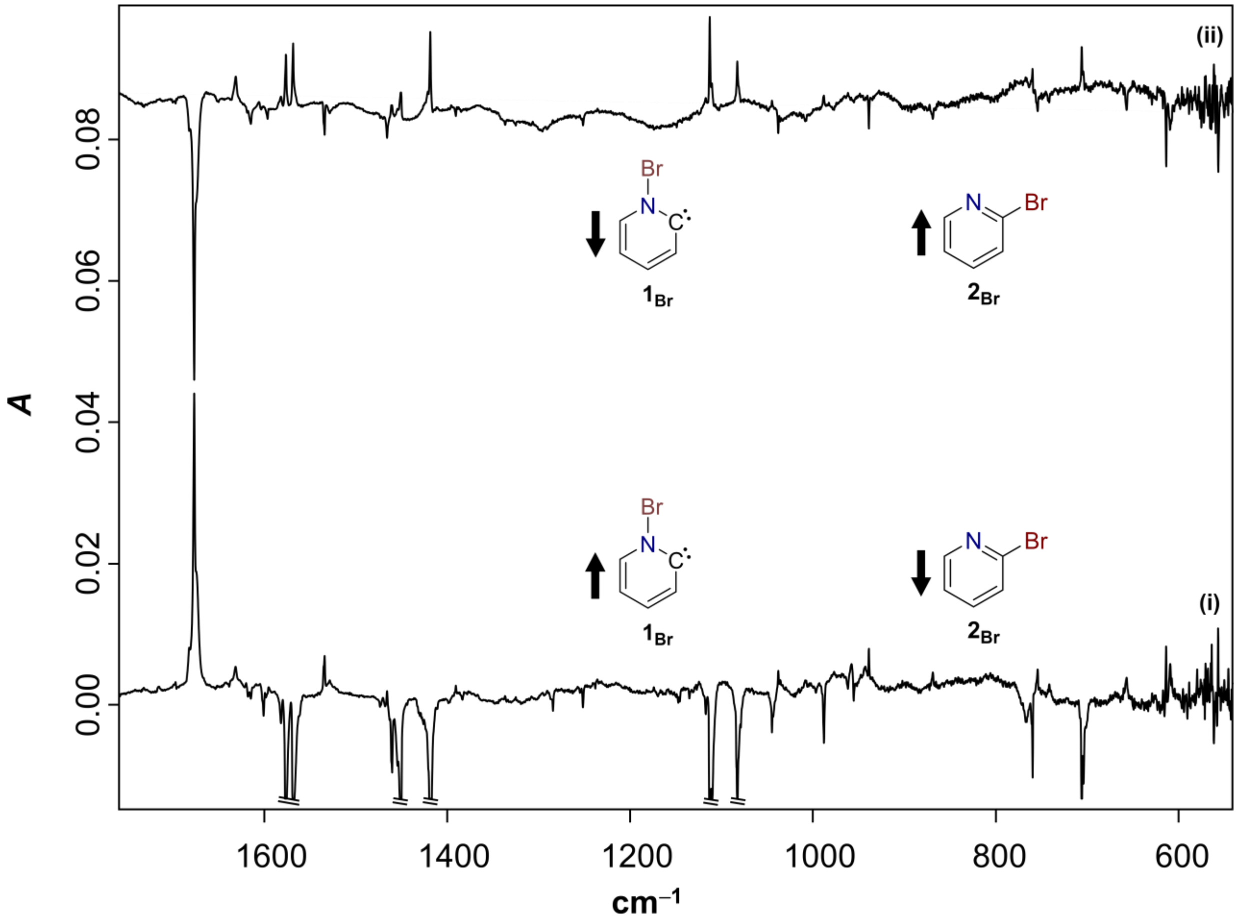

Fig. S1. (i) Difference spectrum after irradiation of the neon matrix containing $\mathbf{2_{Br}}$ for 5 min with λ = 254 nm. (ii) Difference spectrum after exposing the matrix to the room light for 10 min.

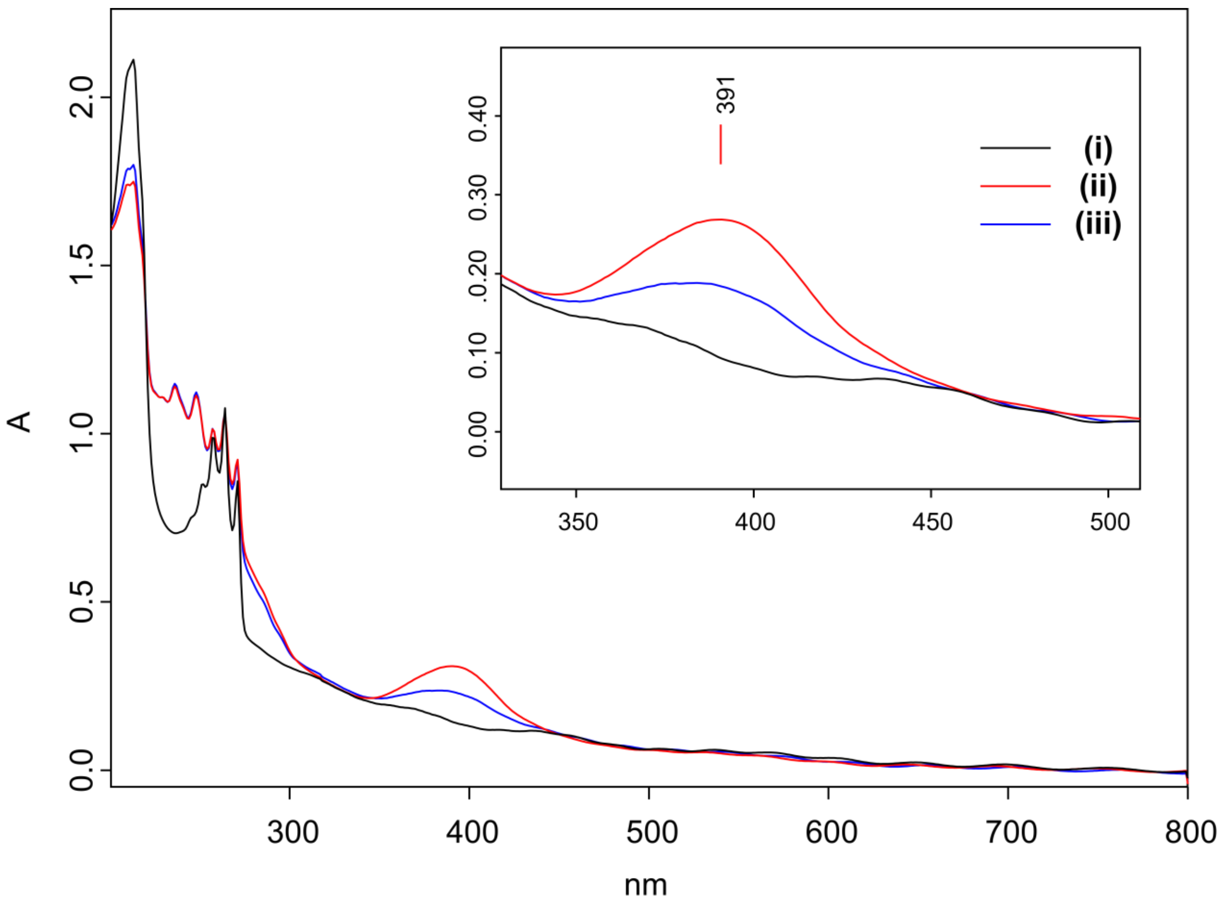

Fig. S2. (i) UV/vis spectrum after co-deposition of precursor $\mathbf{2}_{\mathbf{Br}}$ with a large excess of argon for 4 min. (ii) Irradiation of the matrix for 5 min with λ = 254 nm. (iii) UV/vis spectrum after exposing the matrix to the room light for 5 min.

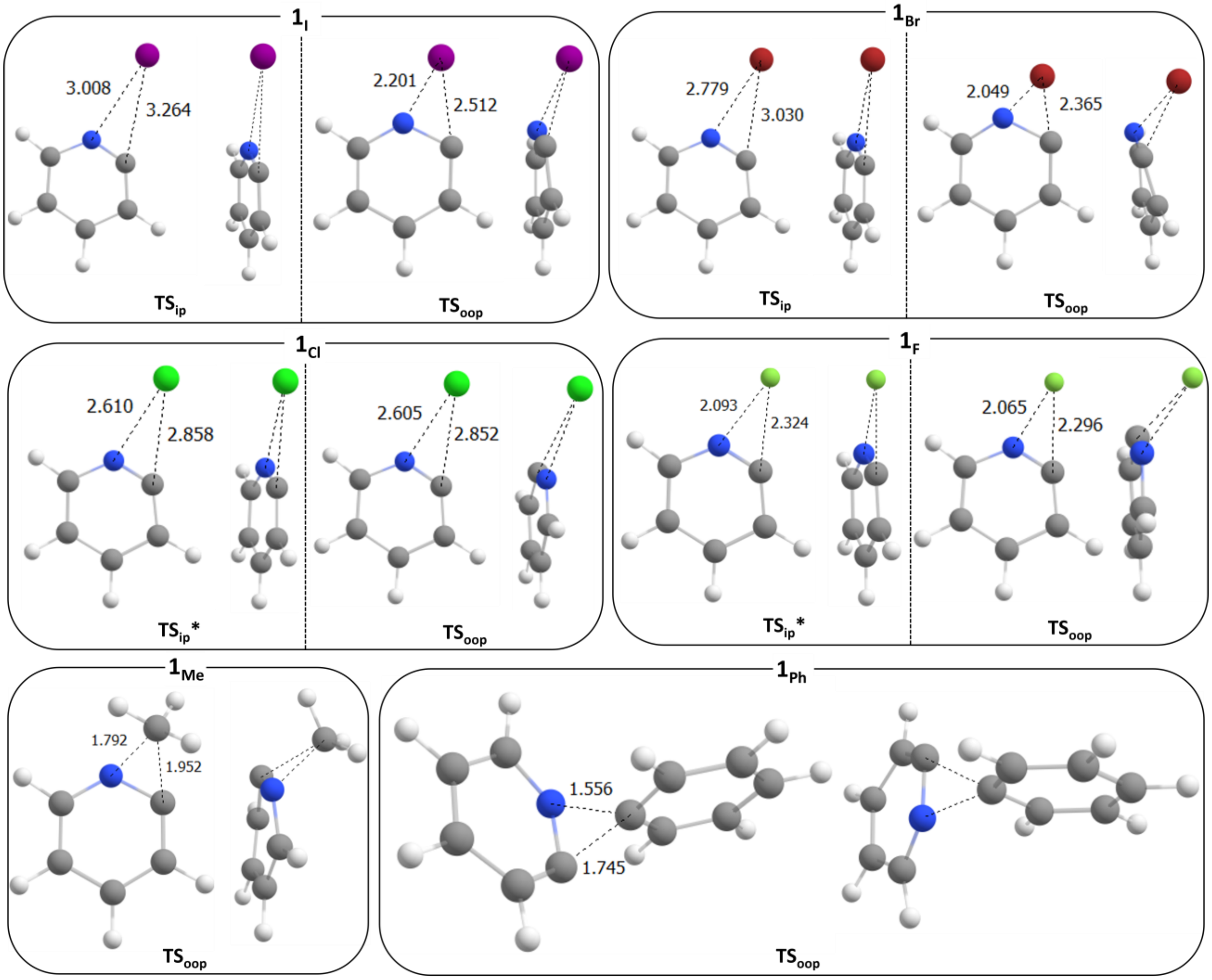

Fig. S3. Two distinct types of saddle points, **TS_{oop}** and **TS_{ip}**, calculated for the [1,2]X (X = I, Br, Cl, and F) shift reactions of the different Hammick intermediates (**1_I**, **1_{Br}**, **1_{Cl}**, and **1_F**). *indicates a second-order saddle point.

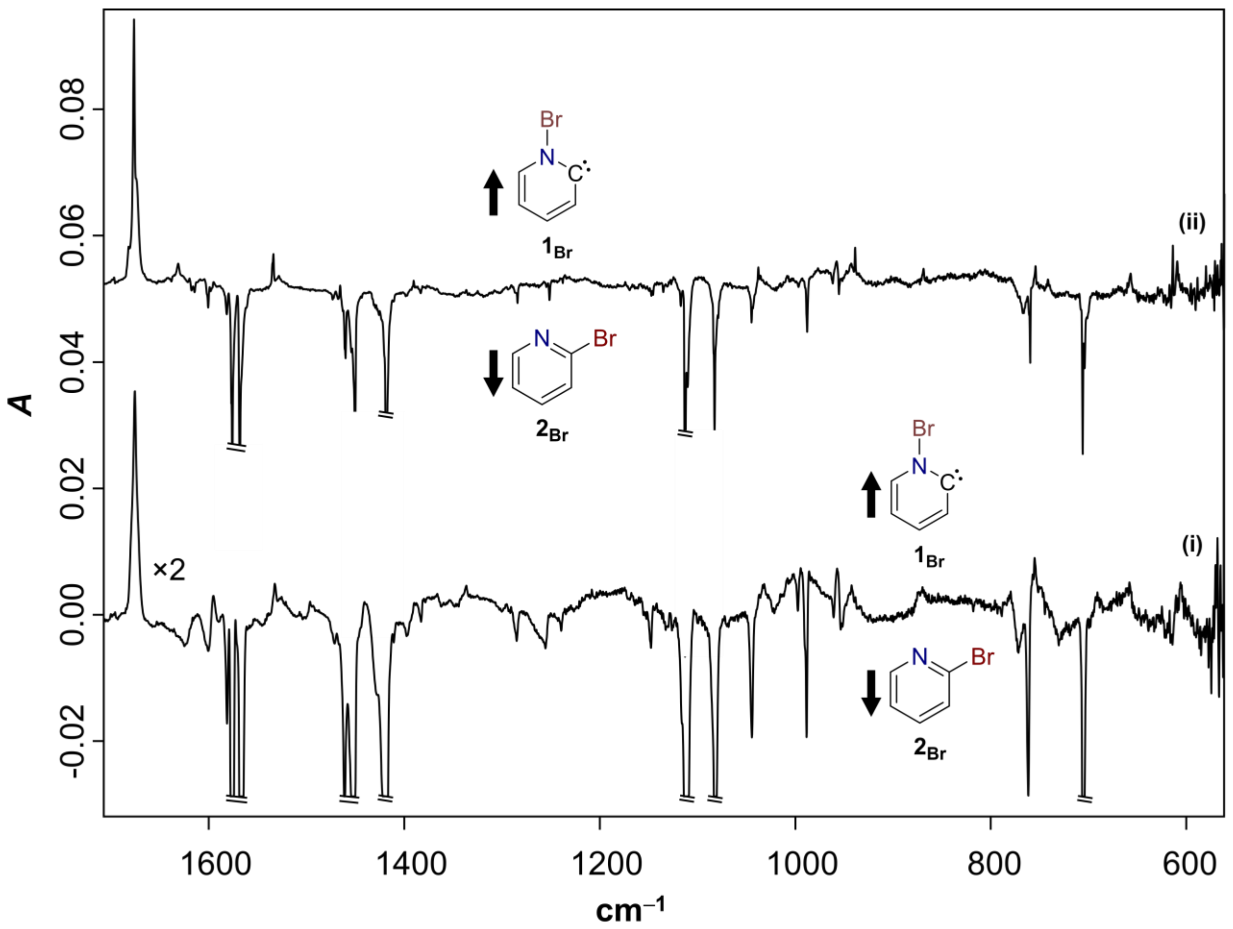

Fig. S4. (i) Difference IR spectrum after irradiation of a neon matrix, which is doped with 3% H_2 and contains **2**$_{Br}$, for 5 min with λ = 254 nm. (ii) Difference IR spectrum after irradiation of a neon matrix containing **2**$_{Br}$ for 5 min with λ = 254 nm.

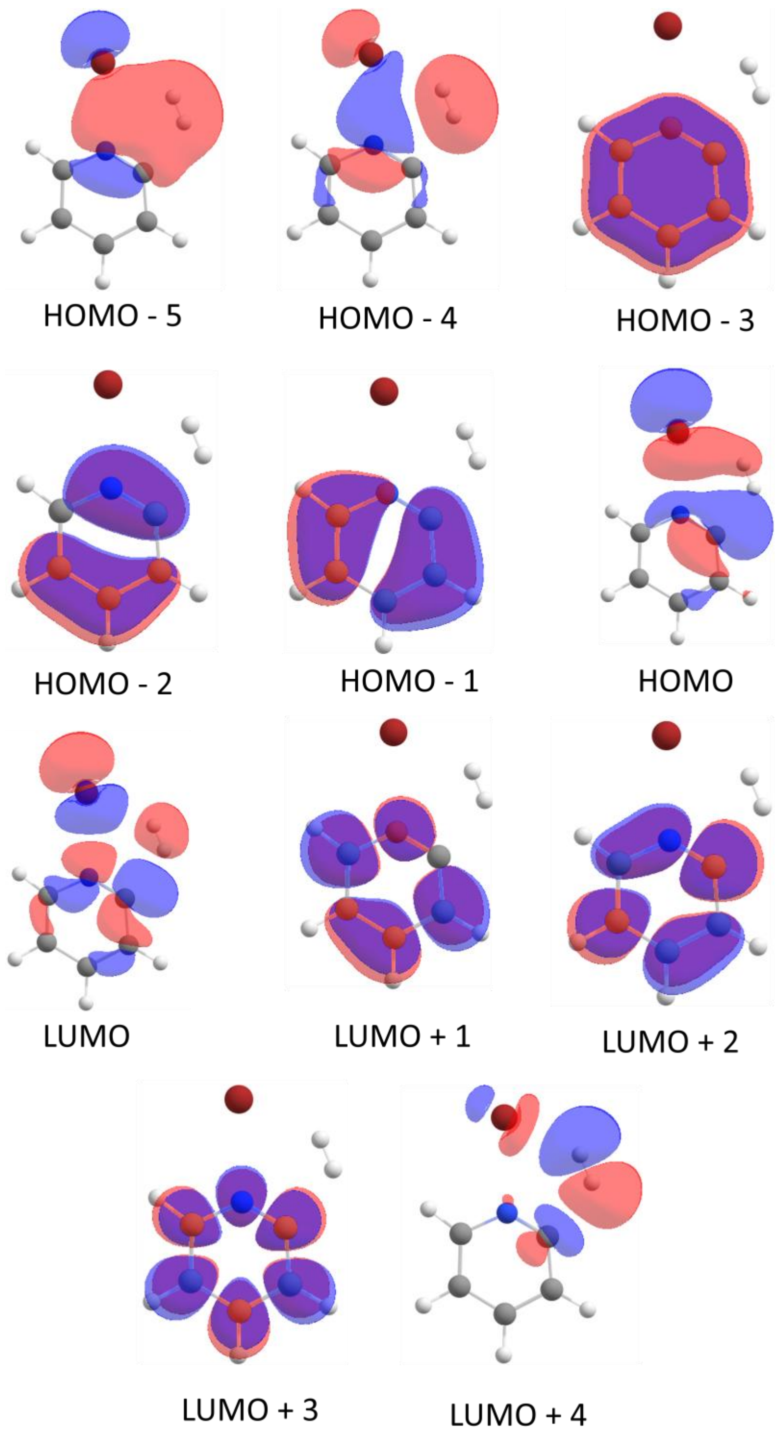

Fig. S5. Active space orbitals used for the transition state calculation corresponding to the H_2 activation by Hammick intermediate $\mathbf{1}_{\mathbf{Br}}$, calculated at the NEVPT2/def2-TZVPP//B2PLYP-D3/def2-TZVPP level of theory. The orbital designations are given in respect to the dominant configuration of the CASSCF wavefunction.

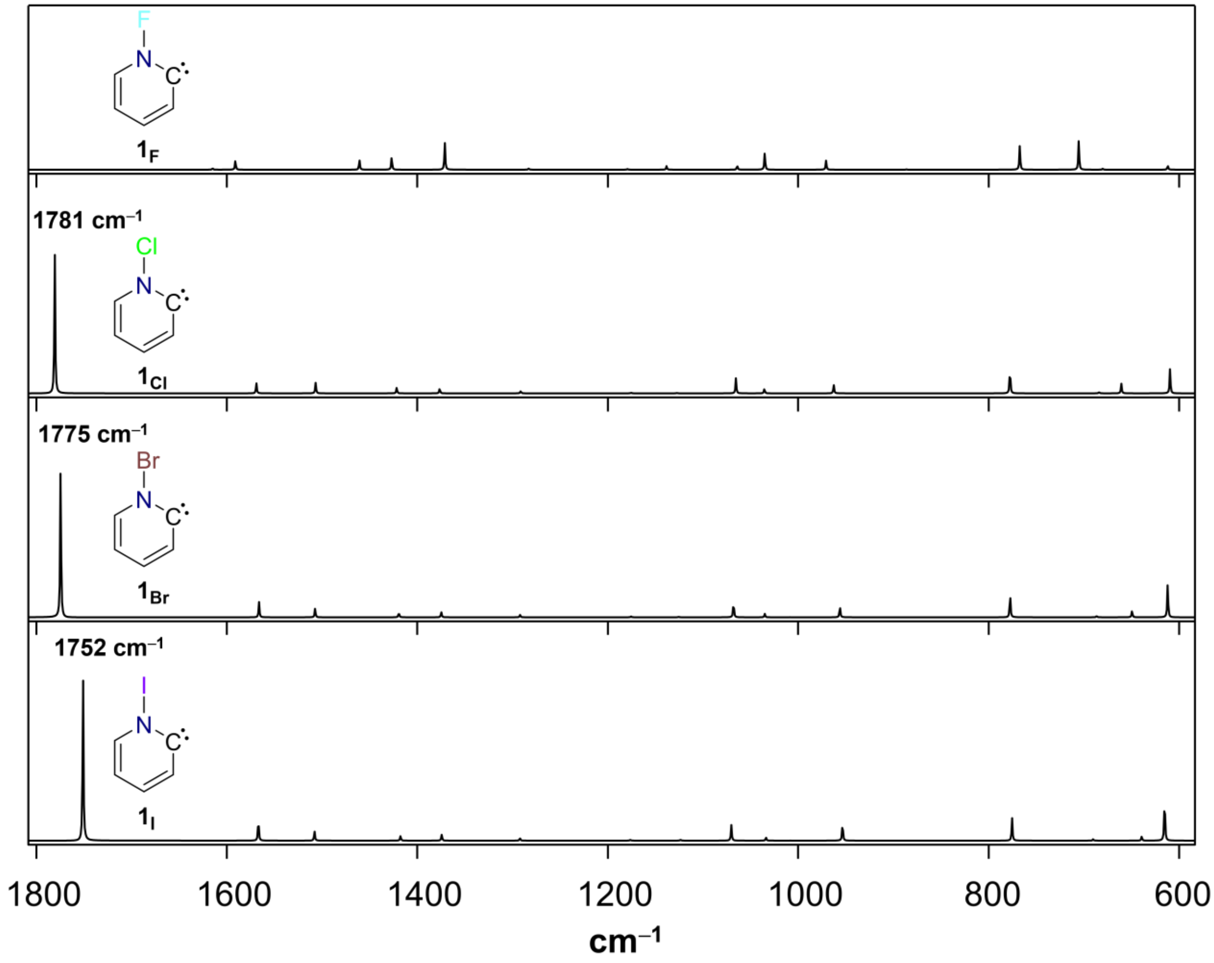

Fig. S6. Comparison of the computed harmonic IR spectra of the different Hammick intermediates **1**$_{I}$, **1**$_{Br}$, **1**$_{Cl}$, and **1**$_{F}$ calculated at the B2PLYP-D3/def2-TZVPP level of theory.

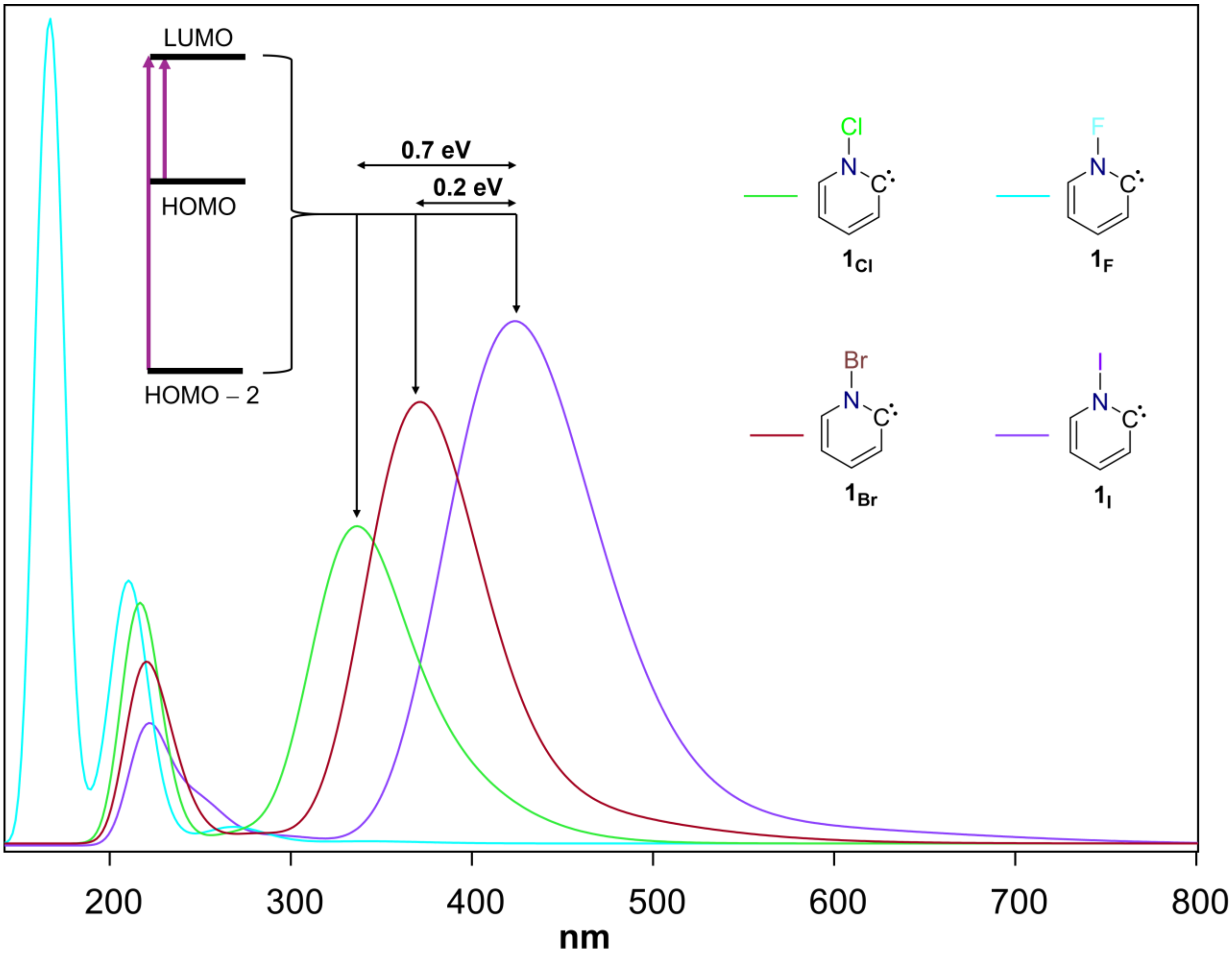

Fig. S7. Comparison of the computed UV/vis spectra of the different Hammick intermediates $\mathbf{1_I}$, $\mathbf{1_{Br}}$, $\mathbf{1_{Cl}}$, and $\mathbf{1_F}$ calculated at the CAM-B3LYP/def2-TZVPP//B2PLYPD3/def2-TZVPP level of theory.

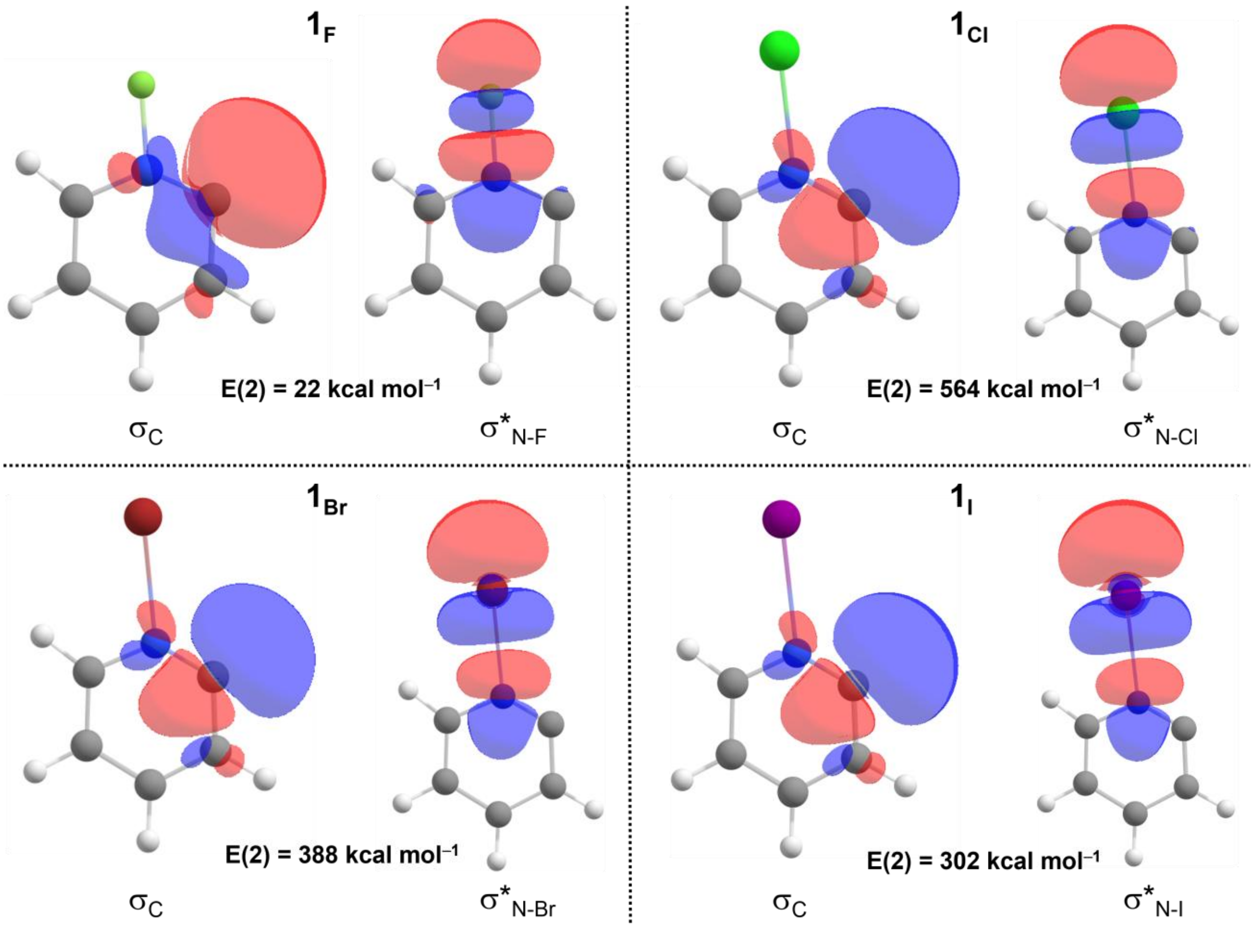

Fig. S8. Natural bond orbitals associated with σ_C and $\sigma^*_{N\text{-}X}$, along with the second-order perturbation interaction (energy *E*(2)) between them, calculated at B3LYP-D3/def2-TZVPP//B2PLYP-D3/def2-TZVPP level of theory for the different Hammick intermediates $\mathbf{1_I}$, $\mathbf{1_{Br}}$, $\mathbf{1_{Cl}}$, and $\mathbf{1_F}$.

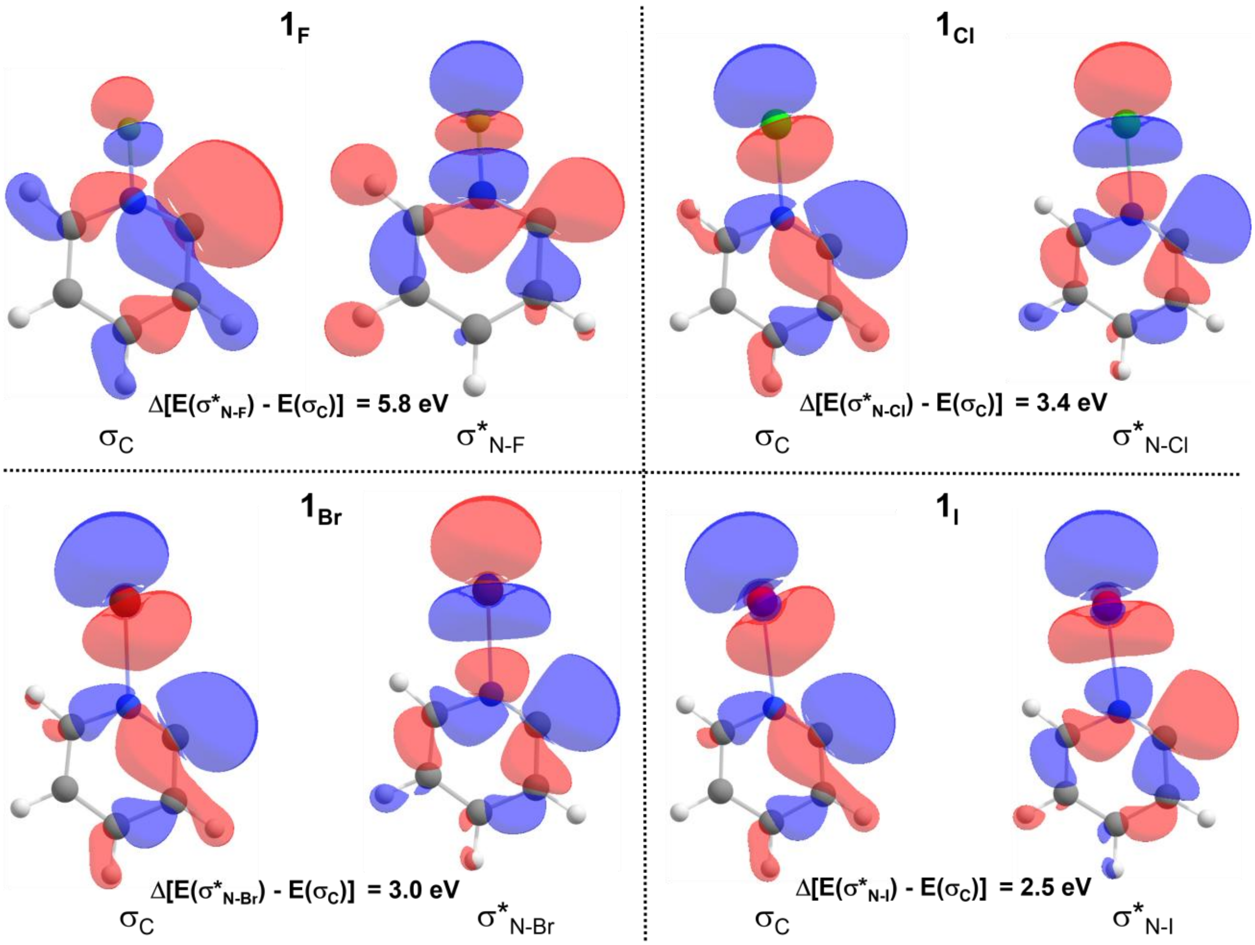

Fig. S9. σ_C and σ^*_{N-X} orbitals, along with their energy gap, calculated at B3LYP-D3/def2-TZVPP//B2PLYP-D3/def2-TZVPP level of theory for different Hammick intermediates **1_I**, **I_{Br}**, **I_{Cl}**, and **1_F**.

IR Band Data

Table S1.
Infrared spectroscopic data of computed (VPT2/B2PLYP-D3/def2-TZVPP level of theory) and observed vibrational frequencies of **1_{Br}** in the pure neon matrix.

Vibrational Mode, ν^a	Experimental		Computed		approx. description
	ν (cm^{-1})	I^b	ν (cm^{-1})	I^c	
27	-	-	3127	3.2	sym. C-H stretch
26	-	-	3111	0.7	sym. C-H stretch
25	-	-	3094	4.5	asym. C-H stretch
24	-	-	3069	3	asym. C-H stretch
23	1677	0.184	1729	100^d	C-N, C-C stretch
22	1534	0.009	1526	4.8	C-C stretch
21	1466	0.003	1474	5.6	C-C stretch
20	1391	0.002	1387	2.5	i.p. C-H bend
19	-	-	1320	2.9	C-C stretch, i.p. C-H bend
18	-	-	1267	1.6	i.p. C-H bend
17	-	-	1162	1	i.p. C-H bend
16	-	-	1109	0.2	i.p. C-H bend
15	1038	0.007	1043	10.5	i.p. C-H bend
14	-	-	1019	2.6	ring breathing
13	-	-	1006	0	o.o.p. C-H bend
12	-	-	954	0	o.o.p. C-H bend
11	939	0.003	940	9.3	C-C-C trigonal bend
10	-	-	884	0	o.o.p. C-H bend
9	754	0.01	766	17.1	o.o.p. C-H bend
8	-	-	679	1.8	C-N-C, C-C-C puckering
7	-	-	642	5.4	i.p. ring deformation
6	614/609	0.008/0.013	604	26.9	i.p. ring deformation
5	-	-	454	0	C-N-C, C-C-C o.o.p. bend

^{a}Numbering of the vibrational modes is taken from the Gaussian output; ^{b}obtained after irradiating the neon matrix containing **2_{Br}** for 5 min; ^{c}intensity relative to the strongest band; ^{d}computed absolute intensity: 303.0 km mol^{-1}.

Cartesian Coordinates and Energies

Table S2.

Cartesian coordinates of optimized geometries calculated at the B2PLYP-D3/def2-TZVPP level of theory. Energies are given in Hartree units at NEVPT2/def2-TZVPP (normal, with ZPVE) and B2PLYP-D3/def2-TZVPP (in parenthesis, with ZPVE).

**1_{Br}**			
-2819.94040 (-2821.17165)			
**C**	1.2815370	2.7109420	0.0000000
**C**	-0.1059080	2.6863500	0.0000000
**C**	-0.6818020	1.4112170	0.0000000
**N**	0.0000000	0.3781920	0.0000000
**C**	1.3449960	0.3032810	0.0000000
**C**	2.0106060	1.5116710	0.0000000
**H**	1.8060550	3.6571270	0.0000000
**H**	-0.6982140	3.5873920	0.0000000
**H**	1.8068260	-0.6701030	0.0000000
**H**	3.0897970	1.5169920	0.0000000
**Br**	-0.8314580	-1.7851290	0.0000000
**TS_{oop} (1_{Br})**			
-2819.86145 (-2821.09824)			
**C**	-2.58660200	-0.47681900	-0.26049400
**C**	-1.56227200	-1.37774200	-0.04114000
**C**	-0.38166000	-0.96172200	0.60424700
**N**	-0.17944400	0.46130800	0.64053500
**C**	-1.06842400	1.33785500	0.11375500
**C**	-2.33408500	0.90780300	-0.19483500
**H**	-3.58146700	-0.82021400	-0.51226200
**H**	-1.69784000	-2.43055600	-0.24439900
**H**	-0.74354900	2.36450900	0.02394400
**H**	-3.11430500	1.62320400	-0.40790300
**Br**	1.65690100	-0.01549600	-0.13349500
**TS_{ip} (1_{Br})**			
-2819.90088 (-2821.14581)			
**C**	-3.04941100	0.52810900	-0.00003000
**C**	-1.98171300	1.42532100	0.00000900
**C**	-0.75989900	0.76999700	0.00004300
**N**	-0.56603800	-0.44011300	0.00004900
**C**	-1.54469100	-1.36185600	0.00001800

**C**	-2.83610500	-0.85928400	-0.00002600
**H**	-4.06048500	0.91279300	-0.00006500
**H**	-2.10369400	2.49529900	0.00000900
**H**	-1.28615600	-2.40859000	0.00002700
**H**	-3.67209900	-1.54247000	-0.00005600
**Br**	2.17473200	0.01743000	-0.00001000
**2_{Br}**			
-2820.03889 (-2821.27133)			
**C**	1.40376000	2.04111700	0.00000000
**C**	1.28774400	0.65911100	0.00000000
**C**	0.00000000	0.12800000	0.00000000
**N**	-1.10738200	0.83952000	0.00000000
**C**	-0.97832800	2.17274900	0.00000000
**C**	0.24953900	2.81856600	0.00000000
**H**	2.38100000	2.50214100	0.00000000
**H**	2.15132100	0.01384700	0.00000000
**H**	-1.90246300	2.73495200	0.00000000
**H**	0.29888500	3.89685200	0.00000000
**Br**	-0.19866700	-1.76976300	0.00000000
**1_I**			
-544.43254 (-544.94955)			
**C**	0.73518000	3.42150600	0.00000000
**C**	-0.61164800	3.08620100	0.00000000
**C**	-0.88911900	1.71628100	0.00000000
**N**	0.00000000	0.84539200	0.00000000
**C**	1.32751700	1.08877900	0.00000000
**C**	1.71064000	2.41384600	0.00000000
**H**	1.03546900	4.46099900	0.00000000
**H**	-1.39010200	3.83255900	0.00000000
**H**	2.00071400	0.24653400	0.00000000
**H**	2.76212300	2.65716600	0.00000000
**I**	-0.34044600	-1.65046600	0.00000000
**TS_{oop} (1_I)**			
-544.37754 (-544.89772)			
**C**	-3.13526300	-0.47213700	-0.15192700
**C**	-2.09420400	-1.38409600	-0.01991400
**C**	-0.80838600	-0.95480100	0.32796700
**N**	-0.62868700	0.44177300	0.35524900
**C**	-1.58534200	1.33631100	0.06705200
**C**	-2.88418600	0.90212600	-0.11561400

**H**	-4.14938000	-0.82051700	-0.29820600
**H**	-2.28051700	-2.44244100	-0.13687000
**H**	-1.29263900	2.37556000	0.02286700
**H**	-3.68247600	1.61932700	-0.23394700
**I**	1.48773800	-0.00733500	-0.04690500
**TS_{ip} (1_I)**			
-544.40348 (-544.92504)			
**C**	2.25415700	1.84019800	0.00000000
**C**	1.20358100	0.93276300	0.00000000
**C**	-0.49333200	2.44632300	0.00000000
**C**	0.44201300	3.46804900	0.00000000
**C**	1.81131000	3.16176700	0.00000000
**H**	3.29566000	1.56520700	0.00000000
**H**	-1.56226500	2.58983000	0.00000000
**H**	0.10891600	4.49508400	0.00000000
**H**	2.53854200	3.96274500	0.00000000
**N**	0.00000000	1.19469600	0.00000000
**I**	-0.67334400	-1.73717600	0.00000000
**2_I**			
-544.51810 (-545.03824)			
**C**	1.23371300	1.23124200	0.00000000
**C**	0.00000000	0.58179500	0.00000000
**C**	-1.16004200	2.53428300	0.00000000
**C**	0.00479500	3.28847300	0.00000000
**C**	1.22428100	2.61873500	0.00000000
**H**	2.15506400	0.67119600	0.00000000
**H**	-2.13130700	3.01070500	0.00000000
**H**	-0.04350400	4.36692600	0.00000000
**H**	2.15590700	3.16641400	0.00000000
**N**	-1.16719300	1.19484000	0.00000000
**I**	-0.03362800	-1.53030700	0.00000000
**1_{Cl}**			
-707.03139 (-707.47086)			
**C**	2.33120400	0.06403400	-0.00000300
**C**	1.74334800	-1.19220700	-0.00000200
**C**	0.34192000	-1.20786500	0.00000400
**N**	-0.30807800	-0.15736200	0.00000300
**C**	0.15944200	1.10406900	0.00000100
**C**	1.53389000	1.21977400	0.00000000
**H**	3.40886800	0.15781000	-0.00000300

**H**	2.32679400	-2.09913300	-0.00000100
**H**	-0.54512700	1.91846900	0.00000100
**H**	1.97847200	2.20305700	0.00000200
**Cl**	-2.45125200	-0.05914700	-0.00000100
**TS_{oop} (1_{Cl})**			
-706.99584 (-707.44323)			
**C**	2.29758700	-0.51875600	-0.14489900
**C**	1.25338800	-1.42508300	0.04832500
**C**	0.04293800	-0.77292300	0.20953400
**N**	-0.15687100	0.43202200	0.23906500
**C**	0.79873300	1.36355500	0.08859100
**C**	2.07588800	0.86676500	-0.12493200
**H**	3.29597500	-0.89705900	-0.31861100
**H**	1.38357700	-2.49387000	0.05164800
**H**	0.53676600	2.40798500	0.13888100
**H**	2.89458400	1.55473900	-0.27307900
**Cl**	-2.69552900	-0.03984100	-0.10188300
**TS_{ip} (1_{Cl})**			
-706.99556 (-707.44322)			
**C**	-2.00473700	-1.28490300	0.00000000
**C**	-0.69652700	-1.76951100	0.00000000
**C**	0.22786000	-0.73727700	0.00000000
**N**	0.00000000	0.46308200	0.00000000
**C**	-1.22932400	1.00446100	0.00000000
**C**	-2.27384100	0.09305800	0.00000000
**H**	-2.82625700	-1.98862500	0.00000000
**H**	-0.44890600	-2.81744700	0.00000000
**H**	-1.33859100	2.07696500	0.00000000
**H**	-3.29190100	0.45239500	0.00000000
**Cl**	2.57441600	0.89412800	0.00000000
**2_{Cl}**			
-707.14078 (-707.58299)			
**C**	-1.57799500	1.17667700	0.00000000
**C**	-0.19213700	1.20704200	-0.00000100
**C**	0.47478900	-0.01627800	0.00000000
**N**	-0.11659700	-1.19408300	0.00000000
**C**	-1.45538800	-1.20532900	0.00000000
**C**	-2.22868100	-0.05351200	0.00000000
**H**	-2.13988300	2.09952700	0.00000100
**H**	0.36044200	2.13282400	-0.00000100

**H**	-1.91707300	-2.18361600	0.00000000
**H**	-3.30602200	-0.11926800	0.00000100
**Cl**	2.21736400	-0.01308700	0.00000000
**1_F**			
-346.87869 (-347.19146)			
**C**	-1.81374000	0.03700400	0.00000100
**C**	-1.17061600	-1.18545500	0.00000100
**C**	0.24639000	-1.28141700	0.00000000
**N**	0.79953500	-0.13127700	-0.00000100
**C**	0.30934400	1.12501200	0.00000000
**C**	-1.05920000	1.22145200	0.00000000
**H**	-2.89364000	0.09193100	0.00000200
**H**	-1.74810000	-2.09851100	0.00000200
**H**	1.00277200	1.94847300	-0.00000100
**H**	-1.52287800	2.19495400	0.00000000
**F**	2.27689200	-0.07972000	-0.00000200
**TS_{oop} (1_F)**			
-346.84640 (-347.15548)			
**C**	-1.73377800	-0.48823000	0.20627600
**C**	-0.73025200	-1.41827700	-0.10038300
**C**	0.47535300	-0.78498600	-0.30810900
**N**	0.69471200	0.41133400	-0.35921900
**C**	-0.21982300	1.36483100	-0.15252800
**C**	-1.48543100	0.88868900	0.17863600
**H**	-2.71508100	-0.84966200	0.48158400
**H**	-0.88807100	-2.48264000	-0.12290500
**H**	0.05125900	2.40309400	-0.25021800
**H**	-2.27161800	1.59265800	0.40553200
**F**	2.56934600	-0.10166100	0.33968900
**TS_{ip} (1_F)**			
-346.82792 (-347.15480)			
**C**	0.51260700	-1.79139800	0.00000000
**C**	-0.81814400	-1.37076100	0.00000000
**C**	-0.91951500	0.01070900	0.00000000
**N**	0.00000000	0.80921200	0.00000000
**C**	1.30162500	0.49341500	0.00000000
**C**	1.56983500	-0.86810200	0.00000000
**H**	0.73065200	-2.85071600	0.00000000
**H**	-1.65509700	-2.04833500	0.00000000
**H**	2.04465300	1.27447400	0.00000000

**H**	2.59469100	-1.20738800	0.00000000
**F**	-1.51037300	2.25825600	0.00000000
**2_F**			
-347.02501 (-347.33205)			
**C**	-1.14024500	1.18200300	0.00000000
**C**	0.24494400	1.20735700	0.00000000
**C**	0.89120800	-0.02333700	-0.00000200
**N**	0.30971200	-1.19492500	0.00000000
**C**	-1.03096200	-1.20215300	0.00000100
**C**	-1.79716100	-0.04702000	0.00000000
**H**	-1.69936100	2.10631300	0.00000100
**H**	0.81218000	2.12447400	0.00000000
**H**	-1.49738000	-2.17791400	0.00000100
**H**	-2.87456600	-0.10699000	0.00000100
**F**	2.23160500	-0.03139000	0.00000100
**$TS_{H2}(1_{Br})$**			
-2821.09629 (-2822.32490)			
**C**	0.24834800	3.09948100	0.00000000
**C**	-1.02610300	2.53875700	0.00000000
**C**	-1.01864500	1.14808900	0.00000000
**N**	0.00000000	0.44881100	0.00000000
**C**	1.25590100	0.90848700	0.00000000
**C**	1.39283300	2.28638500	0.00000000
**H**	0.35801900	4.17557000	0.00000000
**H**	-1.92551100	3.13311300	0.00000000
**H**	2.06483800	0.19380400	0.00000000
**H**	2.37897400	2.72513500	0.00000000
**Br**	-0.10586100	-2.06105300	0.00000000
**H**	-2.27012400	-0.18447000	0.00000000
**H**	-2.01508300	-0.93516300	0.00000000
**3_{Br}**			
-2821.23786 (-2822.46354)			
**C**	-3.33612400	0.00000100	-0.00000400
**C**	-2.63713000	-1.20303100	-0.00000100
**C**	-1.25339200	-1.16657600	0.00000300
**N**	-0.60724100	-0.00000100	0.00000500
**C**	-1.25339000	1.16657500	0.00000300
**C**	-2.63712800	1.20303200	-0.00000100
**H**	-4.41615700	0.00000300	-0.00000700
**H**	-3.15189300	-2.15089600	-0.00000300

**H**	-0.62803400	2.04738800	0.00000500
**H**	-3.15188900	2.15089900	-0.00000300
**Br**	2.35318700	0.00000000	-0.00000100
**H**	-0.62803800	-2.04739100	0.00000400
**H**	0.56814200	-0.00000200	0.00000500
**$TS_{H2}(1_I)$**			
-545.59303 (-546.10673)			
**C**	-0.83568100	3.07721200	0.00000000
**C**	-0.95944400	1.69042100	0.00000000
**C**	1.29069700	1.26812700	0.00000000
**C**	1.54613700	2.62687600	0.00000000
**C**	0.47738600	3.53524500	0.00000000
**H**	-1.68257400	3.74471500	0.00000000
**H**	2.04066100	0.49161900	0.00000000
**H**	2.56761200	2.97541900	0.00000000
**H**	0.67645700	4.59861900	0.00000000
**N**	0.00000000	0.89858900	0.00000000
**I**	-0.15222800	-1.72297400	0.00000000
**H**	-2.38051100	0.39830900	0.00000000
**H**	-2.26813400	-0.36847400	0.00000000
**3_I**			
-545.71804 (-546.22904)			
**C**	0.00000000	0.00000000	-3.89726800
**C**	0.00000000	1.20439800	-3.19974700
**C**	0.00000000	1.17092900	-1.81697900
**N**	0.00000000	0.00000000	-1.17783700
**C**	0.00000000	-1.17092900	-1.81697900
**C**	0.00000000	-1.20439800	-3.19974700
**H**	0.00000000	0.00000000	-4.97725000
**H**	0.00000000	2.15152700	-3.71553900
**H**	0.00000000	2.04711100	-1.18528700
**H**	0.00000000	-2.04711100	-1.18528700
**H**	0.00000000	-2.15152700	-3.71553900
**H**	0.00000000	0.00000000	-0.05727900
**I**	0.00000000	0.00000000	2.01255400

References

[1] S. Grimme, F. Neese, *J. Chem. Phys.* **2007**, *127*. https://doi.org/10.1063/1.2772854

[2] S. Grimme, J. Antony, S. Ehrlich, H. Krieg, *J. Chem. Phys.* **2010**, *132*. https://doi.org/10.1063/1.3382344

[3] F. Weigend, R. Ahlrichs, *Phys. Chem. Chem. Phys.* **2005**, *7*, 3297-3305. http://dx.doi.org/10.1039/B508541A

[4] M. K. Kesharwani, B. Brauer, J. M. L. Martin, *J. Phys. Chem. A* **2015**, *119*, 1701-1714. https://doi.org/10.1021/jp508422u

[5] J. Bloino, V. Barone, *J. Chem. Phys.* **2012**, *136*. https://doi.org/10.1063/1.3695210

[6] C. Angeli, R. Cimiraglia, J.-P. Malrieu, *J. Chem. Phys.* **2002**, *117*, 9138-9153. https://doi.org/10.1063/1.1515317

[7] M. J. Frisch, G. W. Trucks, H. B. Schlegel, G. E. Scuseria, M. A. Robb, J. R. Cheeseman, G. Scalmani, V. Barone, G. A. Petersson, H. Nakatsuji, X. Li, M. Caricato, A. V. Marenich, J. Bloino, B. G. Janesko, R. Gomperts, B. Mennucci, H. P. Hratchian, J. V. Ortiz, A. F. Izmaylov, J. L. Sonnenberg, Williams, F. Ding, F. Lipparini, F. Egidi, J. Goings, B. Peng, A. Petrone, T. Henderson, D. Ranasinghe, V. G. Zakrzewski, J. Gao, N. Rega, G. Zheng, W. Liang, M. Hada, M. Ehara, K. Toyota, R. Fukuda, J. Hasegawa, M. Ishida, T. Nakajima, Y. Honda, O. Kitao, H. Nakai, T. Vreven, K. Throssell, J. A. Montgomery Jr., J. E. Peralta, F. Ogliaro, M. J. Bearpark, J. J. Heyd, E. N. Brothers, K. N. Kudin, V. N. Staroverov, T. A. Keith, R. Kobayashi, J. Normand, K. Raghavachari, A. P. Rendell, J. C. Burant, S. S. Iyengar, J. Tomasi, M. Cossi, J. M. Millam, M. Klene, C. Adamo, R. Cammi, J. W. Ochterski, R. L. Martin, K. Morokuma, O. Farkas, J. B. Foresman, D. J. Fox, Wallingford, CT, **2016**.

[8] F. Neese, *WIREs Comput. Mol. Sci.* **2022**, *12*, e1606. https://wires.onlinelibrary.wiley.com/doi/abs/10.1002/wcms.1606

[9] E. D. Glendening, C. R. Landis, F. Weinhold, *J. Comput. Chem.* **2013**, *34*, 1429-1437. https://onlinelibrary.wiley.com/doi/abs/10.1002/jcc.23266
